# SARS-CoV2 in public spaces in West London, UK during COVID-19 pandemic

**DOI:** 10.1136/bmjresp-2022-001574

**Published:** 2023-05-18

**Authors:** Hisham Abubakar-Waziri, Gopinath Kalaiarasan, Rebecca Wawman, Faye Hobbs, Ian Adcock, Claire Dilliway, Fangxin Fang, Christopher Pain, Alexandra Porter, Pankaj K Bhavsar, Emma Ransome, Vincent Savolainen, Prashant Kumar, Kian Fan Chung

**Affiliations:** 1 Airway Disease, National Heart & Lung Institute, Imperial College London, London, UK; 2 Department of Civil and Environmental Engineering, Global Centre for Clean Air Research, Surrey, UK

**Keywords:** COVID-19, infection control, respiratory infection

## Abstract

**Background:**

Spread of SARS-CoV2 by aerosol is considered an important mode of transmission over distances >2 m, particularly indoors.

**Objectives:**

We determined whether SARS-CoV2 could be detected in the air of enclosed/semi-enclosed public spaces.

**Methods and analysis:**

Between March 2021 and December 2021 during the easing of COVID-19 pandemic restrictions after a period of lockdown, we used total suspended and size-segregated particulate matter (PM) samplers for the detection of SARS-CoV2 in hospitals wards and waiting areas, on public transport, in a university campus and in a primary school in West London.

**Results:**

We collected 207 samples, of which 20 (9.7%) were positive for SARS-CoV2 using quantitative PCR. Positive samples were collected from hospital patient waiting areas, from hospital wards treating patients with COVID-19 using stationary samplers and from train carriages in London underground using personal samplers. Mean virus concentrations varied between 429 500 copies/m^3^ in the hospital emergency waiting area and the more frequent 164 000 copies/m^3^ found in other areas. There were more frequent positive samples from PM samplers in the PM2.5 fractions compared with PM10 and PM1. Culture on Vero cells of all collected samples gave negative results.

**Conclusion:**

During a period of partial opening during the COVID-19 pandemic in London, we detected SARS-CoV2 RNA in the air of hospital waiting areas and wards and of London Underground train carriage. More research is needed to determine the transmission potential of SARS-CoV2 detected in the air.

**WHAT IS ALREADY KNOWN ON THIS TOPIC**

- An important mode of transmission of SARS-CoV-2 apart from droplet inhalation is via virus-laden aerosols (particle size ≤5 µM) supported by reports of its detection in the air under certain conditions.

**WHAT THIS STUDY ADDS**

- SARS-CoV-2 in air samples collected from hospital environments and public spaces such as the London Underground during the partial lifting of restrictions after national lockdown were measured.
- SARS-CoV2 was detected in the air particularly in association with particulate matter size of ≤2.5 µM.

**HOW THIS STUDY MIGHT AFFECT RESEARCH, PRACTICE OR POLICY**

- Air sampling of particles in polluted areas of various public spaces should be monitored for SARS-CoV2 during high levels of infectivity to determine its transmission potential.
- Wearing of face masks during such periods of high infectivity would be recommended, particularly in enclosed or semi-enclosed spaces.

## Introduction

COVID-19 is an acute respiratory disease caused by the novel SARS-CoV2.^1^ Since its identification in Wuhan, China, in 2019,^1^ SARS-CoV-2 has infected 434 million people and caused 5.9 million deaths worldwide, as of February 2022 (WHO, 2022).^2^ Improving our understanding of the characteristics and behaviour of this coronavirus which make it highly transmissible is key to developing future mitigation measures to limit its transmission.

The route of transmission via large droplets (size >5–10 µM) is well-recognised and preventive measures have focused on minimising exposure to these respiratory droplets. Reducing contact with infected persons and contaminated surfaces has been encouraged by implementing the use of face-coverings alongside social distancing and hygiene practices to mitigate against fomite transmission, a potential route of spread.^3^ Transmission via virus-laden aerosols of particle size ≤5 μM in diameter may also be an important mode of transmission over distances of >2 m, that can lead to rapid, wide-scale disease spread.^4^ Airborne transmission of SARS-CoV-2, prompted by directed airflow from ventilation systems, may have caused higher than expected rates of virus spread in a Guangzhou restaurant^5^ and onboard a cruise ship,^6^ despite the usual protective measures being in place. Hamner *et al*^7^ have suggested that aerosols generated by an infected singer during a choir practice transmitted the infection to 32 others in the vicinity.

Under controlled temperature and humidity conditions, aerosolised SARS-CoV-2 is viable and retains infectivity for 3–16 hours.^8 9^ Clusters of SARS-CoV-2 RNA have been reported in aerosols collected from air samples in multiple indoor and outdoor settings,^10–12^ and studies by Lednicky *et al* captured viable SARS-CoV2 virus in air samples by culture on Vero E6 cells.^13 14^ Therefore, we (i) characterised the distribution of SARS-CoV-2 on airborne particles in hospital and other public environments, (ii) assessed the infectivity of SARS-CoV-2 collected from air samples and (iii) compared the performance of a range of air samplers that collect total or size-fractionated particulates.

## Methods

### Sampling sites

Air samples were collected between 10 March 2021 and 14 December 2021 starting from four major hospitals (Charing Cross Hospital, Chelsea and Westminster Hospital, Royal Brompton Hospital and the Royal Marsden Hospital) in intensive care units (ICUs) with adults suffering from COVID-19, respiratory wards and public waiting areas. Collection in ICUs was to test the likelihood of collecting the virus from the air particularly when there was patients with COVID-19 being treated in these rooms. As lockdown measures were gradually lifted after the third national lockdown in England which commenced on 6 January 2021, we sampled in public or semi-public settings from Paddington Rail station, Paddington Underground station (Bakerloo line), inside London Underground carriages, Imperial College University campus and Hampstead Garden Suburb Primary School. Sampling frequency was based on obtaining permissions and being granted access to the sampling locations while sampling frequency was dependent on sampling device used and the capacity to supervise sampling equipment where necessary.

### Air sampling instruments and postsampling processing

A range of sampling instruments was used to collect air particles and liquid bioaerosols (table 1). These included liquid-based total suspended particulate (TSP) samplers, size-segregated particulate matter (PM) samplers and also portable samplers. All samples were stored in Petri dishes and transported on ice to a containment level 2 (CL2) laboratory for processing. The sampling instruments and the processing of the polyurethane foam, Teflon and gelatin filters are described in the online supplemental file 1

10.1136/bmjresp-2022-001574.supp1 Supplementary data

**Table 1:** Air samplers and filter types used

Sampler	Size-segregated particulate samplers				
	Flow rate (L/min)	PM size fraction (μm)	Limit of detection	Filter/Collection medium	Picture
Mini volume sampler	5	<2.5	0.004 µg/m^3^	PTFE	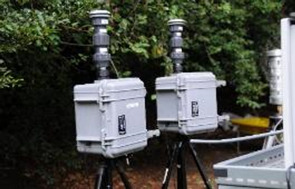
Harvard impactors	30	10 (>2.5 ≤10) 2.5 (≥0.1 <2.5) 0.1 (<0.1)	0.002 µg/m^3^	PUFs and PTFE	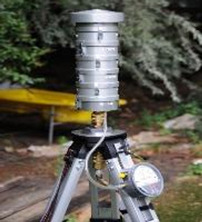
Sioutas cascade impactor	9		0.004 µg/m^3^	PTFE	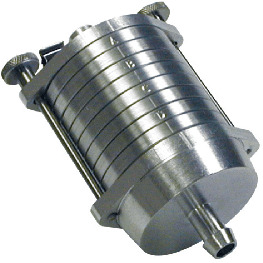
	**TSP samplers**				
Coriolis µ sampler Dimensions—(L×W×D) (36 cm×22 cm×33 cm)	100–300	TSP	>0.5 µm	Phosphate buffered saline	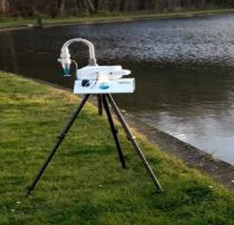
Bio Spot-VIVAS-Bioaerosol Sampler Dimensions—(L×W×D) (76 cm×45.7 cm×37 cm)	8	TSP	5 nm to >10 µm	Water, buffer genomic preservative or nutrient (yeast) broth	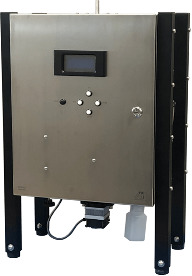
MD8 airport portable air sampler Dimensions—(L×W×D) (135 mm×300 mm×165 mm)	30–125 L/min (adjustable air flow rates)	TSP	>0.65 µm	80 mm gelatin filters (water soluble)	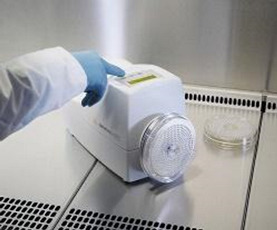
	**Portable Samplers**				
SKC Button sampler This sampler can be deployed as personal sampler	4 L/min	TSP 2.5	0.004 µg/m^3^	25 mm gelatin filter (water soluble PTFE)	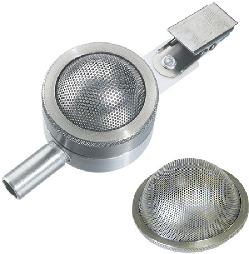

PM, particulate matter; PTFE, polytetrafluoroethylene; PUFs, polyurethane foams; TSP, total suspended particulate.

### RNA extraction and RT-qPCR for SARS-CoV2

Viral RNA extraction and reverse transcription quantitative PCR (RT-qPCR) was performed in a CL2 laboratory. Viral RNA was extracted from 140 μL of sample solution using the QIAamp Viral RNA mini-Kit (Qiagen, Hilden, Germany), following manufacturer’s instructions, and eluted in 35 μL. Extracted Viral RNA from each sample underwent RT-qPCR targeting the n-gene (table 2).

**Table 2:** SARS-CoV-2 primers used for detection of SARS-CoV-2 RNA by RT-qPCR

Oligonucleotide	Sequence 5’>3’ (position)	Reference
N gene Taq1	TCTGGTAAAGGCCAACAACAA (28992)	HSL
N gene Taq2	TGTATGCTTTAGTGGCAGTACG (29073)	HSL
N gene Probe	(6FAM)CTGTCACTAAGAAATCTGCTGCTGAGGC(BHQ1) (29023)	HSL
RNaseP Taq1	AGATTTGGACCTGCGAGCG	Emery *et al*^36^
RNaseP Taq2	GAGCGGCTGTCTCCACAAGT	Emery *et al*^36^
RNaseP Probe	(Cyanine5)TTCTGACCTGAAGGCTCTGCGCG(BHQ2)	Emery *et al*^36^

HSL, Health Services Laboratories; RT-qPCR, reverse transcription quantitative PCR.

To calculate the viral copy number, we simultaneously ran an eight-fold serial dilution of RNA extracted from the research reagent for SARS-CoV-2 RNA (National Institute for Biological Standards and Controls (NIBSC) 19/304) alongside RNA extracted from environmental air samples in a qPCR reaction targeting the N-gene of SARS-CoV-2, with RNase P as an internal control. All samples were run in duplicate. We then calculated the viral copy number per qPCR reaction and subsequently copy number per nanogram of RNA in environmental air samples. Viral copy number (log10) was estimated from Ct values of environmental air samples using the standard curve generated from the qPCR, the research reagent for SARS-CoV-2 RNA (NIBSC 19/304). This value was antilogged and the average divided by the total RNA per qPCR reaction to give the SARS-CoV-2 copy number per nanogram of RNA. The following equation was used to calculate the total virus copy number: virus copy number per ng RNA×total RNA per sample.

The concentration of virus in the air was calculated by dividing the estimated total virus copy number by the total volume of air sampled per cubic metre and expressed as virus copy number per m^3^. The volume of air sampled was calculated by multiplying the sampling rate with the sampling time.

All samples were run in duplicate alongside nuclease-free water as a non-template control and an NIBSC standard 19/304 as a positive control.

### Culture on Vero E6 cells

Vero E6 cells were cultured in a containment level 3 laboratory at 37°C/5% CO_2_ in Dulbecco’s modified eagle medium (DMEM) with 10% fetal bovine serum in T75 flasks. Once at 70% confluency, cultured Vero E6 cells were added to a 24-well plate (20 000 cells/well) and inoculated with extracted air samples in a limiting dilution,^15^ with the addition of DMEM supplemented with 8× penicillin, streptomycin and amphotericin B. Culture plates were incubated at 37°C/5% CO_2_ for a maximum of 6 days and assessed every 24 hours for cytopathic effects using a microscope. If disruption to the cell monolayer was observed during the incubation period, the relevant well was scraped and 100 μL of cells and media placed in 300 μ/µL DNA/RNA shield to inactivate the sample. The samples were then extracted using the Viral Magbead Kit protocol on the Opentrons robot and 1 μL of template tested for the N1 and E gene using qPCR (Roche Master Hydrolysis Probes kit and the Roche 480 Lightcycler machine) performed according to manufacturer’s instructions.

### Patient and public involvement

There has been no patient or public involvement.

## Results

**Figure 1:**
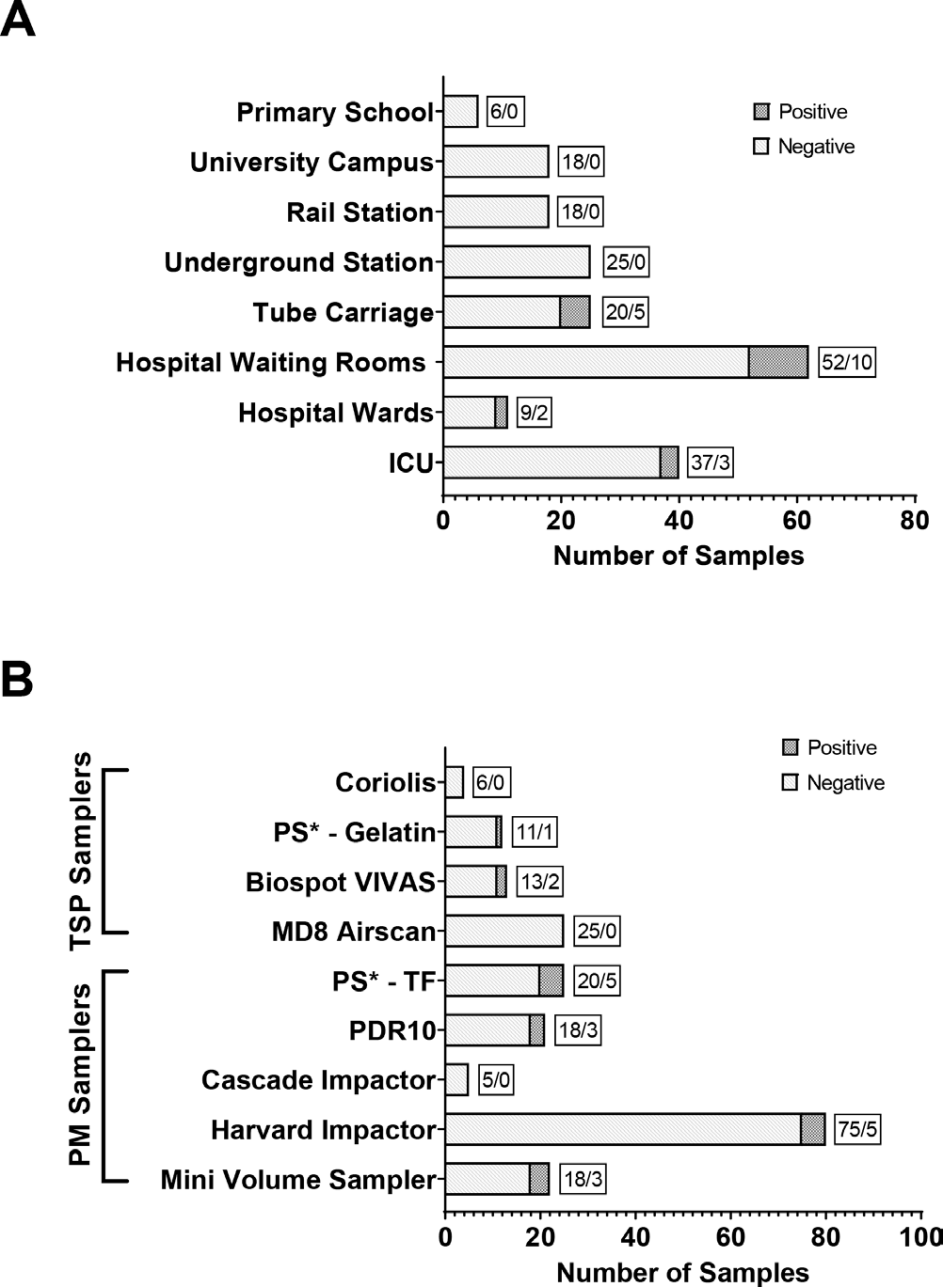
Number of SARS-CoV-2 RNA-positive (solid) and SARS-CoV-2 RNA-negative (open) samples collected at each sampling site (panel A) and using individual air samplers (panel B). The text box represents the ratio of SARS-CoV-2-negative and SARS-CoV-2-positive samples (negative/positive). ICU, intensive care unit; PM, particulate matter; TSP, total suspended particulate.

In total, 207 samples were collected across all sites over 8 months using eight sampling instruments (table 3). Of these, 20 (9.7%) samples tested positive for detectable SARS-CoV-2 RNA, as summarised in table 4. Most of the positive samples were collected in hospital environments (n=15), from ‘lower-risk’ publicly accessible communal waiting areas (n=10) and from ‘higher-risk’ hospital rooms with a confirmed SARS-CoV-2-positive in-patient on ICUs and respiratory wards (n=5). The remaining five positive samples were from a London Underground train carriage, collected using a portable sampler on two separate London Underground journeys. The remaining 187 samples were negative on PCR testing. Figure 1 summarises the total number of collected samples at each site, using each instrument, together with the SARS-CoV-2-positive samples. The only situation where SARS-CoV2 infection was confirmed was in the patients treated in ICUs. We did not check for the presence of SARS-CoV2 infection in any of the other sites where we sampled the air.

**Table 3:** Samples collected using each air sampler, sampling location and SARS-CoV-2 RNA-positive samples

	PM samplers				TSP			
	Mini volume sampler	Harvard impactor	Coriolis cascade impactor	pDR1500	MD8 Sartorius	Biospot VIVAS	SKC	Coriolis
	Number of samples							
Charing Cross Hospital adult intensive care unit	2	4	0	2	10	2	3	0
Charing Cross Hospital general ward	0	0	0	0	3	1	0	0
Charing Cross Hospital emergency department waiting room	2	8	0	1	0	0	0	0
Chelsea and Westminster Hospital general respiratory ward	0	0	0	0	2	3	2	0
Chelsea and Westminster Hospital emergency department waiting room	2	8	0	2	0	0	0	0
Royal Brompton Hospital adult intensive care unit	0	0	0	0	10	3	4	0
Royal Brompton Hospital outpatient waiting room	4	16	0	4	0	0	0	2
Royal Marsden Oncology medical day unit	2	8	0	3	0	0	0	0
Paddington Rail station (information boards)	3	12	0	3	0	0	0	0
Paddington Underground station (ticket hall)	1	4	0	1	0	2	0	0
Paddington Underground escalators	3	12	0	2	0	1	0	0
Paddington Underground platform (Bakerloo)	0	0	0	0	0	0	0	0
London Underground train carriage	0	0	0	0	0	0	25	0
University campus—Eastside Bar	1	0	5	1	0	0	3	2
University campus—RSM Workshop	1	4	0	1	0	1	0	0
Primary school	1	4	0	1	0	0	0	0
Total samples	22	80	5	21	25	13	37	4
Total positive	4	5	0	3	0	2	6	0

PM, particulate matter; TSP, total suspended particulate.

**Table 4:** PCR-positive SARS-CoV2 RNA samples

Sample ID	Sampler type	Filter type	Site	Date of collection (lockdown measure)*	Seven-day average COVID-19 cases in area†	Volume air sampled (m^3^)	Copy no./Volume air (m^3^)	PM2.5 (μg/m^3^) (mean±SD)
**CV30**	Biospot VIVAS	Condensation	NP room on respiratory ward	25 March 2021 (step 1)	12.7	0.96	899	NA
**CV40**	SKC Button sampler	Gelatin	Respiratory ward	17 April 2021 (step 2)	9.9	0.12	64 741	NA
**CV46**	Harvard impactor	PM2.5 ≥0.1 <2.5 µm	Medical day unit	12 May 2021 (step 2)	5	43.2	533	1.1±0.3
**CV47‡**	Harvard impactor	PM10 >2.5 ≤10 µm	Medical day unit	12 May 2021 (step 2)	5	43.2	23 559	
**CV48‡**	MVS	Teflon 2.5	Medical day unit	12 May 2021 (step 2)	5	7.2	286 434	
**CV54**	Biospot VIVAS	Condensation	ICU	26 May 2021 (step 3)	7.9	0.96	17 174	1.8±1.0
**CV55**	MVS	Teflon 2.5	ICU	26 May 2021 (step 3)	7.9	7.2	1254	
**CV56**	PDR10	Teflon 2.5	ICU	26 May 2021 (step 3)	7.9	2.88	225 022	
**CV67**	MVS	Teflon 2.5	ED waiting room	02 June 2021 (step 3)	13.7	7.2	151 809	6.9±2.4
**CV68**	PDR10	Teflon 2.5	ED waiting room	02 June 2021 (step 3)	13.7	7.2	707 284	
**CV110**	Button sampler	Teflon	Northern Line (Archway-Tottenham Court Road) 20 min	02 August 2021 (step 3)	140.9	0.12	80 936	NA
**CV112**	Button sampler	Teflon	Piccadilly Line (Leicester Sq-Gloucester Rd) 20 min	02 August 2021 (step 3)	83.0	0.12	159 269	NA
**CV114**	Button sampler	Teflon	Northern Line (Kennington-Leicester Square) 20 min	02 August 2021 (step 3)	171.7	0.12	147 161	NA
**CV116**	Harvard impactor	PM10 >2.5 ≤10 µm	Medical day unit	30 November 2021 (no restrictions)	107.6	43.2	158	1.4±1.9
**CV117**	Harvard impactor	PM2.5 ≥0.1 <2.5 µm	Medical day unit	30 November 2021 (no restrictions)	107.6	43.2	118	
**CV118**	Harvard impactor	PM0.1 <0.1 µm	Medical day unit	30 November 2021	107.6	43.2	513	
**CV120**	MVS	Teflon	Medical day unit	30 November 2021 (no restrictions)	107.6	7.2	709	
**CV121**	PDR (GMF)	Microglass	Medical day unit	30 November 2021 (no restrictions)	107.6	2.88	7092	
**CV122**	Button sampler	Teflon	Northern Line 30 min (Archway-Leicester’s Square)	17 August 2021 (no restrictions)	148.4	0.12	65 862	NA
**CV123**	Button sampler	Teflon	Piccadilly Line 30 min (Leicester’s Square-South Kensington)	17 August 2021 (no restrictions)	79.6	0.12	143 892	NA

*UK government four-step roadmap for lifting restrictions and mapping a route back to normal life (https://www.gov.uk/government/publications/covid-19-response-spring-2021): step 1—travel prohibited, work from home, schools resume, limited university return, outdoors gatherings and sports permitted but limited to six people. Step 2—re-opening of non-essential retail, personal care premises (hairdressers/nail salons), public buildings (libraries, community centres), leisure facilities. Step 3—gatherings of up to 30 people permitted, re-opening of indoor entertainment venues, hotels, hostels, indoor events with capacity of 1000 people permitted. Step 4—gradual re-opening of nightclubs, eased restrictions on large events, outdoor meeting encouraged.

†Seven-day average number of COVID-19 cases were obtained from the coronavirus data dashboard (https://coronavirus.data.gov.uk/details/cases) developed by the UK Health Security Agency.

‡Samples CV47 and CV48 were successfully sequenced and found to be associated with the S-variant B1.1.7. Spike P681H detected was associated with a Nigerian mutation (N gene R203K, G204R, S235F, D288G).

ED, emergency department; ICU, intensive care unit; MVS, mini volume sampler; NA, not available; NP, negative pressure; PM, particulate matter.

### Hospital wards

One hundred and thirteen samples were collected across four hospitals in ICUs, respiratory wards and communal waiting areas. Forty samples were collected in negative pressure rooms in the ICU in two hospitals, 8 using PM samplers (mini volume sampler (MVS) and PDR10) and 32 using TSP collectors (Sartorius MD8, gelatin-loaded button sampler and Biospot VIVAS). All three positive samples were collected in the same sampling session using the MVS, PDR10 and Biospot VIVAS, in the room of a conscious patient breathing high-flow oxygen.

A total of 11 samples were obtained from respiratory wards using TSP collectors (MD8, Biospot VIVAS and gelatin-loaded SKC Button sampler), including one negative pressure room and two ambient pressure rooms. Two positive samples were obtained from two different locations, one in an ambient pressure room using the gelatin-loaded SKC Button sampler, with the other obtained in a negative pressure room using the Biospot VIVAS. Although both rooms were from different hospital sites, they both housed self-ventilating patients wearing a mask for supplementary oxygen.

### Communal hospital areas

Sixty-two samples were collected from four communal waiting areas across four hospitals. Most samples (60) were collected using size-fractionated PM samplers (Harvard impactors, MVS, PDR10) and 2 from TSP collectors. Twenty-three samples were collected in emergency department (ED) ED waiting rooms, 26 in an outpatient waiting area and 13 in a chemotherapy day unit (CDU). Ten samples were positive in total, eight from two separate sampling sessions in the CDU and two from a general hospital ED waiting area. All positive samples were collected using size-fractionated PM samplers.

### London Underground and railway station

Sixty-nine samples were collected using the Harvard impactors, cascade impactor, MVS, PDR and VIVAS from high-footfall areas of public transport including 18 from a major railway station, 26 in the ticket hall, near escalators and on the platform of a London Underground station and 25 from inside a deep-line London Underground train carriage using a portable sampling pump attached to polytetrafluoroethylene (PTFE)-loaded filter cassettes. All samples collected from the railway and underground stations were negative on PCR. Of the 25 samples taken inside the train carriage, 5 were positive for SARS-CoV-2 RNA. These were collected from two separate journeys with the personal sampler worn by the same person.

All 28 samples taken from a university campus, including 11 from a busy university bar and 7 from a university engineering workshop were negative. All six samples collected in a primary school were also negative.

### Airborne virus concentrations in different environments

**Figure 2:**
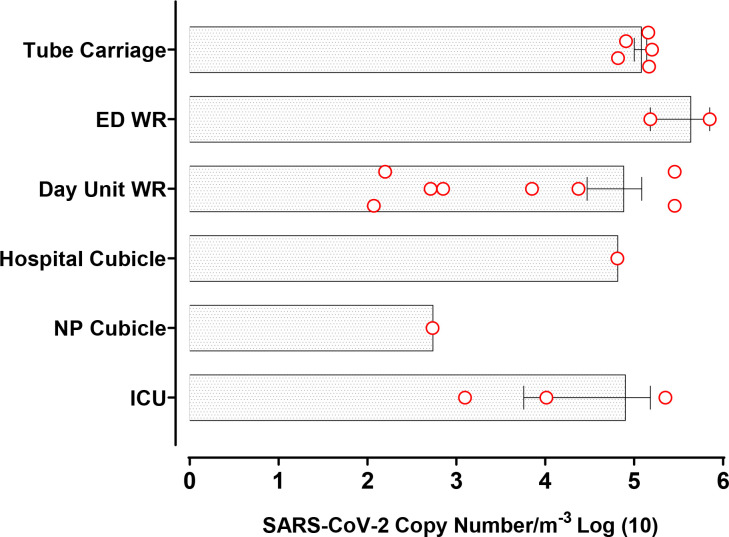
Mean SARS-CoV-2 RNA copy numbers/m^3^ in hospital environments and on public transport, using all air samplers. Each circle represents a single measure of the SARS-CoV-2 RNA copy number detected within a given location. ED, emergency department; ICU, intensive care unit; NP, negative pressure; WR, waiting room.

Mean virus concentrations across different locations were expressed as virus copies/m^3^ (figure 2). The amount of virus collected across all samplers ranged from 118 virus copies/m^3^ of air to 707 284 copies/m^3^, with the higher concentrations of virus surpassing what has been reported previously (table 5). In hospital public areas, these were highest in the ED waiting area (429 500 copies/m^3^) and were markedly higher than mean concentrations measured in the CDU (75 523 copies/m^3^). Moreover, mean virus concentrations were lower on the respiratory ward cubicles: 539 copies/m^3^ in a respiratory ward negative pressure cubicle and 78 850 copies/m^3^ in an ICU negative pressure cubicle. All measurements conducted in hospital cubicles involved a self-ventilating patient who was within 1 week of admission to hospital and had a positive SARS-CoV-2 PCR test within 24 hours of sampling. The second highest mean concentration of virus (119 360 copies/m^3^) was measured inside the London Underground train carriage.

**Table 5:** Maximum detectable SARS-CoV-2 concentrations RNA copies/m^3^ in recent studies

	Maximum detectable SARS-CoV-2 concentration (RNA copies/m^3^)
Zhou *et al*^37^	219
Hu *et al*^17^	11 200
Lednicky *et al*^13^	0.74
Lednicky *et al*^14^	31 400
Chia *et al*^38^	3380
Liu *et al*^39^	113
This study	707 284

Two samples, both collected from the CDU from two separate devices in the same sampling session, underwent successful genomic sequencing to determine the SARS-CoV-2 variant (see table 4). Both were related to the B1.1.7 variant, with one sample demonstrating a spike-protein mutation associated with S-variant SARS-CoV2 lineage B1.1.7.

### Size distribution of particles with positive samples

**Figure 3:**
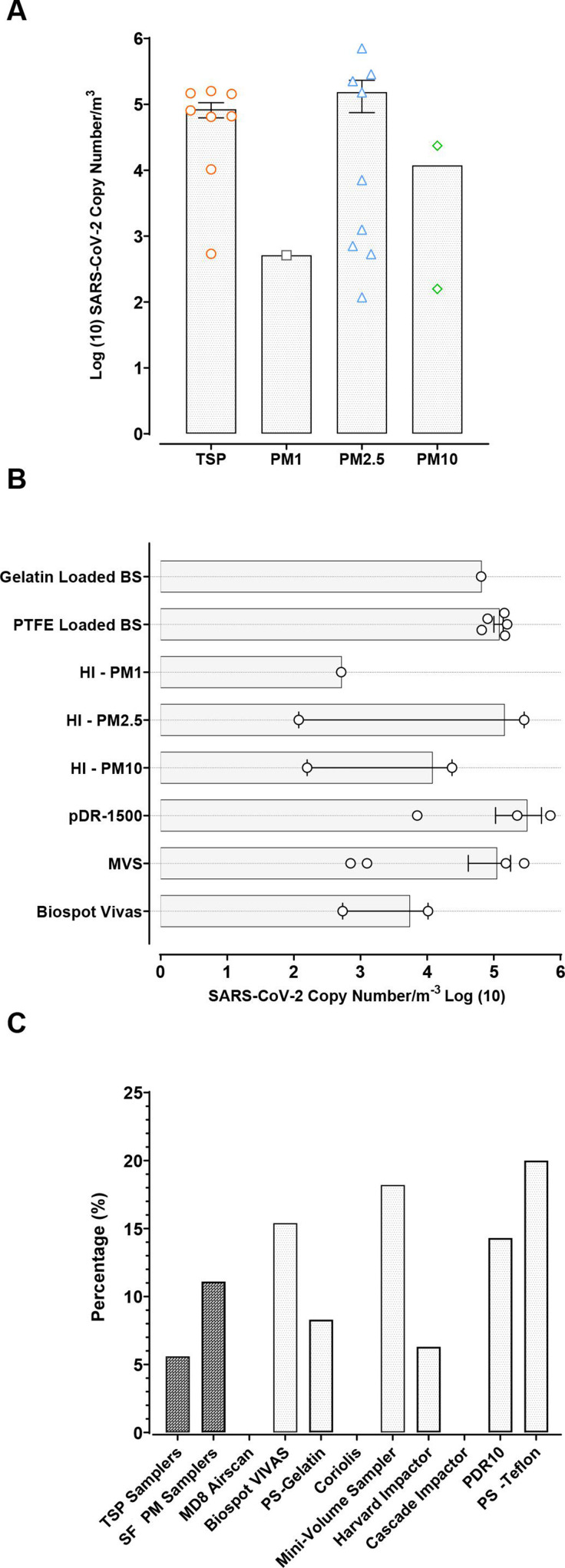
SARS-CoV-2-positive air samples. Panel A demonstrates the mean concentration of virus detected on TSP and on each PM size fraction. Panel B demonstrates the concentration (copies/m^3^) of SARS-CoV-2-positive samples collected using different samplers. Each point represents a single measurement, collected by the indicated instrument sampler. Panel C shows the % pick up of SARS-CoV-2-positive sample for TSP and SF PM samplers and for each individual air sampler. BS, button sampler; HI Harvard impactor; MVS, mini volume sampler; PM, particulate matter; PS, personal sampler; PTFE, polytetrafluoroethylene filter; SF, size fractionated; TSP, total suspended particulate.

SARS-CoV-2 RNA was most frequently detected in PM samplers compared with liquid-based TSP samplers (PM: 12 samples (85%) vs liquid-based TSP: 8 samples (15%)) across all settings (figure 3A). Positive samples collected on PM filters were more frequent in the PM2.5 size fraction (n=10). Using the Harvard sampler that fractionated the particles, two positive samples were detected on PM10 fractions on two different occasions, with positive samples also simultaneously detected in the PM2.5 fraction on both occasions with the PM10 fraction containing a higher amount of virus compared with the PM2.5 fraction. The VIVAS collected the most liquid-based positive samples (n=2) with the only other positive sample coming from a gelatin-loaded button sampler.

The highest concentrations of virus were found on samples collected in the PM2.5 fraction (figure 3A, B). The second highest were found using TSP, with the next highest in the PM10 fraction. The lowest concentrations of virus were found in the PM1 fraction.

### Performance of air samplers in detection of SARS-CoV-2

Out of 207 samples, most were collected using PM samplers (152 samples; 73% of total) compared with liquid TSP samplers (54 samples; 27% of total). PM samplers using PTFE and polyurethane foam filters more frequently detected SARS-CoV-2 (n=17) compared with liquid-based TSP samplers (n=3), with a detection rate of 11% vs 6% (figure 3C).

## Discussion

We detected SARS-CoV-2 RNA in air samples collected from several hospitals and public spaces in London during the partial lifting of restrictions, following the third national lockdown. Most of the positive samples were collected inside hospitals, first in an ICU with patients with COVID-19 receiving treatment and then in respiratory wards, as has been previously reported.^16 17^ In hospital areas, we also picked up the virus in non-COVID-19-associated waiting areas in clinics, in this case, in the waiting area of an oncology clinic. We found the presence of SARS-CoV-2 on airborne PM collected on public transport, in two separate samples from inside two different deep-line London Underground train carriages, using a portable sampler worn by a carriage passenger for 30 min during the rush-hour commute. Interestingly, we did not find virus on the samples collected on the platforms over a longer period of 8 hours. The presence of SARS-CoV2 on airborne PM picked up in the carriage but not on the platforms is of great interest because levels of PM on the London Underground are known to be very high.^18 19^ Previously, SARS-CoV2 virus have been detected from air samples collected between May and July 2020 inside buses and subway trains in Barcelona, Spain, particularly on PM2.5.^20^ We did not pick up virus from the other sites (university campus and primary school), possibly owing to policies in place which encourage regular testing for detection of COVID-19 infection, hence a lower likelihood of infected persons detected earlier and isolation occurring sooner. Negative air samples for the presence of SARS-CoV2 in different indoor public places in Italy has also been reported.^21^ Moreover, we did not detect virus in the railway station, possibly owing to the better ventilation conditions due to more open space and lower particulate concentrations.

We used a wide range of air samplers and sampling techniques because there is no general agreement as to the best method of sampling for SARS-CoV2 detection in the air.^22^ The highest concentrations of SARS-CoV2 virus collected on 7 samples that was above 100 000 copy numbers per m^3^ occasions may have arisen from our longer sampling periods, particularly when we used the size-fractionated PM samplers for up to 8 hours of collection. Surprisingly, we were able to detect the virus after only 30 min while using portable air samplers. We observed higher pick-up rates on size-segregated PM samplers compared with TSP collectors. When assessing size-segregated PM samplers, the highest pick-up rates were seen using the mini volume sampler (18% pick-up rate) and pDR-1500 (14% pick-rate), both of which collect samples at lower flow rates (2–5 L/min) with lower pick up using the Harvard impactor (6%, sample rate of 30 L/min), although a proportionately higher number of samples were collected by the Harvard impactor. As depicted in table 4, on the 2 days when virus was detected in both PM10 (containing PM >2.5 ≤10 µm) and PM2.5 (containing PM ≥0.1 <2.5 µm) fractions from the Harvard impactor as depicted in table 4, the amount of virus was higher in the PM10 fraction compared with the PM2.5 fraction as would be predicted. Interestingly, the next highest pick-up rates were noted in the portable SKC personal sampler (15% pick-up rate), with five out of the six positive samples collected on PTFE filters. Therefore, we were more likely to detect SARS-CoV2 when collecting at lower flow rates using techniques that impact PM and segregate them in terms of size. This higher pick-up rate may reflect the longer sampling periods when using particulate samplers, increasing the sampling volume and likelihood of capturing virus in the air. Alternatively, high flow rates may damage the virus.

Our detection of SARS-CoV-2 on PM, particularly PM2.5 and PM10 supports the notion of an interplay between virus and PM, suggesting there may be an interaction between SARS-CoV2 and PM.^23^ PM has been reported to interact with pathogens and may act as a vector for disease transmission.^24–26^ Most (75%) of our positive samples were collected onto filters which contained PM2.5 fine particles, in agreement with findings of Kayalar *et al* in Turkey.^11^ In the outdoor study performed in Bergamo Italy, samples collected were positive in PM10 samples for SARS-CoV2, because PM10 was the only particulate fraction that was collected.^12^ This link would provide support for the epidemiological studies in China and the USA that indicate that people living in high pollution areas particularly with high concentrations of PM2.5, experience more severe COVID-19 disease with higher mortality rates should they get infected with SARS-CoV2 virus.^27 28^ One possibility is that PM2.5 may act as a conduit for the virus to reach the small airways and the alveoli, thereby favouring the development of pneumonia.^29^ In addition, exposure to PM may upregulate expression of ACE-2, the receptor which the virus binds to via its spike protein,^30^ indicating that PM may also increase susceptibility to SARS-CoV-2 infection. Thus, the COVID-19 pandemic highlights the need for lowering the levels of PM2.5, to limit the spread of SARS-CoV2 spread.^31^

Despite this co-existence of SARS-CoV2 and PM2.5, we did not culture SARS-CoV-2 after inoculating to Vero E6 cells despite some of the high levels of SARS-CoV2 copies observed in many samples. A possible explanation could be a lack of viability of the suspended virus sampled. While the SARS-CoV2 virus may remain viable for up to 3 hours in aerosols generated into air,^9^ a recent study found that there was a rapid loss of infectivity of the aerosols within minutes due to the elevation of pH as the aerosol evaporates.^32^ However, stability of the virus in connection with PM is not known. It has been reported that PM2.5-derived reactive oxygen species resulting from interaction with epithelial cells may impair the structure and survival of influenza A that binds to PM2.5.^33^ It is possible that interactions with PM may alter the survivability of the virus and, therefore, the determination of the minimum concentration of SARS-CoV2 virus associated with PMs to propagate viral replication in Vero E6 cells or lung epithelial cells is needed.

The pick up of positive samples in these public/semi-public places is very much dependent on the presence of any infected asymptomatic or mildly symptomatic persons and producing aerosols containing SARS-CoV2 virus. One limitation of the study is our inability to determine whether there were any positive cases present or circulating within the spaces sampled. This may well explain why we did not detect any SARS-CoV2 in the bars, university campus rooms or in the school that we sampled. In addition to the source of the virus, other factors that will determine the amount and survivability of the virus are the environmental conditions such as temperature, humidity and airflow.^34^ It is unclear as to whether large airborne titres of virus are being produced by multiple infectious people residing in the same space, or from potential ‘super-spreaders’. Moreover, knowing whether an infected case was present, would help us estimate how long respired virus remains airborne.

Another limitation is the use of a single primer targeting the N-gene of SARS-CoV-2 in processed airborne samples, via RT-qPCR. There are data to suggest primers targeting the N-gene is highly sensitive, with increased risk of false positives.^35^ This increases the risk of overestimating the viral copy number. On the other hand, inability to extract 100% of particles from all filter types could mean that virus concentrations have been underestimated, as we were unable to extract the entire sample. In two samples collected, we were able to confirm, by full sequencing, that the variants collected were the dominant variants at that time, that is, the S-variant B1.1.7, that confirm that SARS-CoV2 virus was detected.

In summary, we detected SARS-CoV-2 RNA virus in size-fractionated PM samples, particularly in the fine fractions of PM collected from hospital waiting areas and wards and in London Underground train carriage. This indicates that SARS-CoV2 can circulate in the air, but whether it is active needs further work such as elucidation any potential interactions of PM2.5 with SARS-CoV2 in the air and at the lung epithelial surface. Air sampling, using size-fractionated PM samplers or portable air samplers, may be important in determining the transmission potential of SARS-CoV2. In addition, as a matter of precaution, the wearing of face masks during such periods would be recommended, particularly in indoor and semi-open environments.

## Data Availability

Data sharing not applicable as no datasets generated and/or analysed for this study.
