# Supplementary material for: SARS-CoV2 in public spaces in West London, UK during COVID-19 pandemic
Source: BMJ Open Respir Res. 2023 May 18;10(1):e001574. doi: 10.1136/bmjresp-2022-001574 (PMC10200905; doi:10.1136/bmjresp-2022-001574)
Supplement: Supplementary data [file bmjresp-2022-001574supp001.pdf]

SARS-CoV2 in public spaces in West London UK during COVID-19 pandemic

Hisham Abubakar-Waziri[1], Gopinath Kalaiarasan[2], Rebecca Wawman[1], Faye Hobbs[4], Ian M Adcock[1], Claire Dilliway[5], Fangxin Fang[3], Christopher Pain[5], Alexandra Porter[3], Pankaj Bhavsar[1], Emma Ransome[4], Vincent Savolainen[4], Prashant Kumar[2], Kian Fan Chung[1]

Supplemental materials

Abubakar-Waziri H, *et al. BMJ Open Resp Res* 2023; 10:e001574. doi: 10.1136/bmjresp-2022-001574

Methods

Liquid-based Total Suspended Particulate (TSP) Samplers

Unsegregated total suspended particulates (TSP) were collected using the commercially-available liquid-based samplers including the Biospot-VIVAS (Aerosol Devices Inc., Ft. Collins, CO), the Sartorius MD8 (Sartorius AG, Germany) and the Coriolis µ air sampler (Bertin Technologies). The Biospot VIVAS has been shown to be efficacious in collecting viral particles [11] and airborne particles using a water vapour condensation method, at a rate of 8 litres per minute (L min^{-1}). Air initially passes through a cool temperature conditioner, followed by passage through the initiator at 30°C which coalesces particles as small as 8 nm into larger droplets greater than 2 µm in diameter. The enlarged particles are then collected through a set of nozzles onto 1.5 mL of liquid collection media. The collection media used, composed of 1.5mL 1x phosphate buffered saline (PBS), 0.5% (w/v) bovine albumin fraction V and sucrose added to a final concentration of 0.2M, has been used to successfully culture SARS-CoV-2 [1]. Liquid samples were stored at 4°C and transported on ice for viral RNA extraction and SARS-CoV-2 PCR testing.

The Sartorius MD8 (Sartorius AG, Germany) sampled air at a rate of 30 L min^{-1} for 30 minutes, impacting particles onto a sterile gelatin filter (80 mm diameter, 3 µm pore size, type 80-ACD, Sartorius AG). After sampling, the gelatin filter was transferred aseptically into a Petri dish and transported on ice and stored in a refrigerator at 4°C for later processing.

We also used the Coriolis µ air sampler (Bertin Technologies) which collects air at 100 L min^{-1}. Samples were collected every 30 minutes into a conical vial containing 15 mL phosphate-buffered saline. Samples were stored at 4°C with viral RNA extraction fluid and qRT-PCR performed on neat samples.

Size-segregated Particulate Matter Samplers

Size-segregated particles were collected using a MiniVOL sampler, (Airmetrics, Springfield, OR, USA) Harvard Cascade Impactor (HCCI) and personal DataRAM (pDR-1500). The MiniVOL sampler (MVS) (Airmetrics, Springfield, OR, USA) was equipped with PM_{10} and $PM_{2.5}$ impactors and collects $PM_{2.5}$ particles at a flow rate of 5 L min^{-1} using a double-diaphragm pump with laminar flow valve technology. This means that the PM fraction collected will be ≤2.5 μm. The particles were collected onto a 47 mm polytetrafluoroethylene (PTFE) filter (TISCH Scientific, 201 S Miami Ave, Cleves, OH 45002, USA). Particles were collected on Teflon filters because they are hydrophobic in nature, chemically resistant, have high initial particle capture efficiency across different flow rates and are suitable for gravimetric, chemical and microscopic analysis of PM.

The Harvard Impactor collected size-segregated particles of PM_{10} (coarse), $PM_{2.5}$ (fine) and PM_1 (ultrafine) on polyurethane foams (PUF) (Merry weather Foam, OH, USA) at a rate of 30 L min^{-1} with particles fractionated at three different impaction stages. Particles ≤100 nm (PM0.1) were collected simultaneously onto 47 mm PTFE filters (TISCH Scientific 201 S Miami Ave, Cleves, OH 45002, USA) at the final stage of this cascade impactor. This means that the coarse fraction collected will contain PM >2.5≤10 μm, the fine fraction PM ≥0.1<2.5 μm, and the ultrafine particles below 100nm of PM<0.1 μm.

The personal DataRAM™ (pDR-1500) aerosol monitor (Thermo Scientific, Franklin, MA, USA) is a sensitive nephelometric monitor with a cyclone inlet for measurement and collection of $PM_{2.5}$ particles. The pDR-1500 reported the average $PM_{2.5}$ concentration every 1 min and collected particles onto 37 mm glass fibre filters

through the sensing zone at a flow rate of 1.52 L min^{-1} for a cut size of 2.5 µm diameter. All the filters used in the above-mentioned size segregated particle samplers were allowed to equilibrate in a weighing room with controlled temperature (21°C) and relative humidity (30–40%) for a minimum of 24 hr prior to weighing before and after sampling. After each sampling session, filters were removed using forceps and inserted into sterile Petri dishes and sealed using parafilm. These were transported on ice to a level 2 laboratory for particle extraction and SARS-CoV-2 PCR testing.

Portable Samplers

We also sampled using the portable SKC Button sampler (SKC LTD, Dorset, UK). Air was extracted at a rate of 4 L min^{-1} using the SKC Airchek touch pump, which was attached to the outlet of the button or a filter cassette. loaded with a 37mm PTFE filter. The sampler was attached to an individual's clothing, on their chest, ~2 cm under their clavicle, whilst wearing an FFP3 mask. Devices were calibrated using an adaptor by attaching the Button sampler to the inlet of the button, and the inlet of the calibration adaptor to a HVAC system prior to use. Air samples were collected directly onto a dissolvable 25 mm gelatin filter (1.0µm pore size, SKC LTD, Dorset) when sampling with the button, or unto the 37mm PTFE filter (0.3µm pore size, SKC LTD, Dorset) pre-loaded to a filter cassette, using an appropriate adaptor as per manufacturer's instructions. The button sampler collects 'inhalable' particles with a 1µm-100µm aerodynamic diameter as defined by the British Standards Institution (BS EN 481:1993). The PTFE loaded cassette collects total suspended particulate with an aerodynamic diameter above 0.3µm.

Post-sampling processing

PUF and TF filters

Filters were removed from air samplers using forceps and placed in a Petri dish at the sampling site. PBS was added to the filters in a class II biological safety cabinet (10 ml to polyurethane foam (PUF), 5 ml to PFTE) and sealed with parafilm. This was then placed on a shaker for 30 minutes at 70 RPM to gently transfer particles from the filter into the solution. After 30 minutes, the solution was transferred into a 10 ml Falcon tube in an L2 HEPA-filtered hood using a pipette. 1 ml of the solution was immediately sent for culture on Vero-E6 cells in a 1.5 ml Eppendorf, with the remaining solution stored at -80ºC for RNA extraction and RT-qPCR.

Gelatin filters

80 mm gelatin filters were placed into a Petri dish with 10 ml of PBS added whereas 37 mm gelatin filters from portable samplers were placed in 50 ml falcon tubes with 5 ml of PBS added. They were then placed in a shaker at 70 RPM and warmed to 28ºC for 30 minutes to dissolve the filter. The remaining solution was transferred into a 10 ml Falcon tube in a L2 HEPA-filtered hood using a pipette. 1 ml of the solution was immediately sent for culture on Vero-E6 cells in a 1.5 ml Eppendorf tube, with the remaining solution stored at -80 ºC for RNA extraction and RT-qPCR.

Biospot VIVAS and Coriolis

Air particles from the Biospot VIVAS were directly collected in liquid medium in a small petri dish and conical flask respectively. After sampling, an equal volume of the solution (between 500-1000 µl) was transferred into three separate 1.5ml Eppendorf tubes. One Eppendorf tube containing solution was immediately sent for culture on Vero-E6 cells, with the remaining stored at -80ºC for RNA extraction and RT-qPCR.

Abubakar-Waziri H, *et al. BMJ Open Resp Res* 2023; 10:e001574. doi: 10.1136/bmjresp-2022-001574

Reference:

1. Lednicky JA, Lauzardo M, Fan ZH, *et al.* Viable SARS-CoV-2 in the air of a hospital room with COVID-19 patients. *International Journal of Infectious Diseases* 2020;**100**:476–82. doi:https://doi.org/10.1016/j.ijid.2020.09.025
